# What Does Moral Agency Mean for Nurses in the Era of Artificial Intelligence?

**DOI:** 10.1002/hast.70030

**Published:** 2026-02-04

**Authors:** Connie M. Ulrich, Oonjee Oh, Sang Bin You, Maxim Topaz, Zahra Rahemi, Liz Stokes, Lisiane Pruinelli, George Demiris, Patricia Flatley Brennan

**Keywords:** agent, artificial intelligence systems, moral agency, nursing, nursing ethics, clinical ethics

## Abstract

Being a moral agent was once thought to be an irreplaceable, uniquely human role for nurses and other health care professionals who care for patients and their families during illness and hospitalization. Today, however, artificial intelligence systems are often referred to as “artificial moral agents,” “agentic,” and “autonomous agents.” As these systems begin to function in various capacities within health care organizations and to perform specialized duties, the question arises as to whether the next step will be to replace nurses and other health care professionals as moral agents. Focusing primarily on nurses, this essay explores the concept of moral agency, asking whether it remains exclusive to humans or can be conferred on AI systems. We argue that AI systems should not supplant nurses’ moral agency, as patients come to hospitals or any other health care setting to be heard, seen, and valued by skilled professionals, not to seek care from machines.

## Essay

Nursing, a field long associated with trustworthiness, is one of the many areas of health care being transformed by the use of artificial intelligence technologies. AI applications in clinical nursing include predicting a patient's future state and care outcomes, interpreting clinical images and pathology studies, summarizing information in patient records, drafting clinical messages, and—a quickly emerging function—passively capturing and reporting conversations that occur during patient care encounters. Administrative applications of AI that nurses might encounter include forecasting staffing needs and workload, facilitating preauthorizations for procedures, enabling payments, detecting fraud, generating automated responses to patient queries, and enhancing a facility's resilience to climate change. In nursing research, AI approaches such as machine learning and natural‐language processing aid in literature synthesis, the evaluation of large genomic data resources and other extensive datasets, the development of predictive models for personalized care, the computational testing of evidence‐based interventions, and the optimization of clinical trials.

While some uses of AI may benefit nurses and patients, the intersection of nursing and AI technologies also poses challenges and presents opportunities that require careful consideration. For instance, what are the implications of letting an AI tool determine what was noteworthy among the things a patient or family member told a nurse during an office visit? Can AI be trusted to summarize that encounter in terms that will serve the nurse, their colleagues, and the patient well? How should nurses respond to AI predictions about care outcomes that seem at odds with their own expectations for their patients? Such questions bear on nurses’ crucial role as moral agents.

A *moral agent* is a person who has the ability to discern right from wrong and to be held accountable for their actions.^1^ Nurses are moral agents when they participate in the process of caring for patients and fostering patients’ progress toward healing and health. U.S. data show that nurses have earned the public's trust because of their high ethical standards.^2^ If AI is to be fully useful to nurses, it must be designed so that it is well‐aligned with nurses’ moral agency and so that its use enhances and maintains that much‐valued public trust. This essay explores the meaning of nurses’ moral agency in the era of AI and offers suggestions for actions that nurses can take to ensure the preservation of that agency and their profession's trustworthiness.

## Nurses as Moral Agents

Nurses are the largest group of professionals providing health care, with over five million licensed registered nurses in the United States.^3^ While every nurse who has achieved the profession's pedagogical and didactic requirements and is licensed by the state can legally practice, clinical care is not just technical—it is inherently moral. Nurses, like all health professionals, base their engagement with patients on moral premises.^4^ Every health care professional receives training in the foundational ethical guidelines that outline their professional and ethical obligations to patients, colleagues, and society. Whether in research or clinical care, nurses and other health care professionals must often exercise their practical wisdom, grounded in their practice of virtues or their understanding of other ethics‐related frameworks.^5^ What makes moral agency foundational to nursing are the forms it takes and the way it guides care: nursing is aligned with the patient's values, preferences, and capabilities.^6^

Philosophers have offered several definitions of moral agency, each highlighting different aspects of it. Aristotle, the founding figure of virtue theory, emphasized moral responsibility as what distinguishes moral agents and nonmoral agents. From Aristotle's perspective, a moral agent acts for the sake of their own flourishing and happiness.^7^ Kant, known for his meticulous articulation of deontology, viewed moral agents as sovereigns who legislate their own laws or practical principles that could be universally applied to a community of rational beings without contradiction.^8^ Although moral agents are bound by these self‐imposed laws,^9^ Kant emphasized the autonomy of moral agents, as sovereigns who determine their own laws through reason, independent of external constraints.^10^ By contrast, Hume, one of the early sentimentalists, argued that feelings of sympathy were the basis of morality and viewed self‐regulation of one's own character as a requisite for moral agents.^11^

Aligning with the key concepts introduced by foundational philosophers, the American Association of Colleges of Nurses states that, for moral agency to occur, three elements are essential: awareness of an ethical issue, making a moral choice, and taking moral action on that choice.^12^ The American Nurses Association equates moral agency with moral autonomy, which is the freedom from internal and external constraints that could hinder ethical responsibility and moral action.^13^ The Canadian Nurses Association also defines “moral agent” in the context of nursing as someone who has the ethical responsibility for how they behave and interact with the individuals receiving their care. This association's Code of Ethics for Nurses outlines self‐reflection and dialogue as ways for nurses to recognize their role as moral agents in patient care.^14^

Nurses work in various settings, collaborating with physicians, pharmacists, psychologists, psychiatrists, social workers, dieticians, physical therapists, and others, and their clinical workdays are often characterized by uncertainty, urgency, and vulnerability. At times, nurses must make decisions without clear rules, drawing on their clinical knowledge and ethical judgment to balance scientific evidence and clinical experience with the values of a particular patient and family, as well as with the policies and expectations—spoken and unspoken—of the institutional context in which they work. Such complexity makes nurses, as it does other health care professionals, unique agents, distinct from protocols or algorithms.

Nurses rely on their ethical training and sensitivities to determine which protocols should be followed and what steps should be taken when protocols fall short. AI systems help create treatment protocols, but final application in care delivery depends on a skilled health care professional. Health care professionals must decide when and how to apply an AI‐suggested protocol and when to depart from it to align with patients’ goals of care and to respect patients’ individual preferences and personal dignity. These day‐to‐day situations call for humans’ minds to discern the relevant values that underlie patient‐family‐clinician interactions. Nurses’ moral agency is at the heart of these decisions and actions.

Indeed, as moral agents, nurses carry a core responsibility grounded in fundamental principles of biomedical ethics and codes of ethics. These principles form the ethical foundation of clinical decision‐making: promoting patient well‐being, avoiding harm, respecting patient choice, and ensuring fairness in care delivery.^15^ In practice, these principles are often expressed through values such as compassion, shown in responding meaningfully to suffering; humility, evident when acknowledging one's limits and listening deeply; courage, needed to speak up when care is compromised; and integrity, essential to remaining aligned with ethical commitments even when working under pressure.^16^ While recent advances in generative AI have led to tools, such as chatbots, that may appear empathetic or even outperform humans in perceived compassion, many argue that the moral discernment underpinning these values cannot be fully replicated by algorithms.^17^ Some believe that the human judgment that undergirds these qualities cannot be programmed and therefore cannot be executed by an AI algorithm. They are qualities cultivated through years of human experience, ethical reflection, and human connection that go beyond surface‐level responsiveness.

Responsibility and accountability are fundamental aspects of exercising moral agency. AI systems can be responsible in that a system can make an error, cause harm, and be blamed. However, current technological systems that are powered by AI lack sentience or self‐perception and do not have a human sense of responsibility when harm occurs. Responsible use of AI in a clinical setting lies squarely with the human actor—in this case, the nurse. Thus, it remains to be seen whether AI systems will enhance patient care and support for families and provide trustworthy support for nurses and other health care professionals in their day‐to‐day work or whether such systems will work to erode the sacred relationship between patients and their health care professionals.

## Moral Agency in AI

At present, AI systems are not moral agents. However, as AI technology continues to advance with great speed, generating human‐like responses and potentially developing the ability to self‐correct and to generate rationales for its explanations, commentators debate whether these AI systems could possess moral agency, could act as moral agents, or will be treated as moral agents. These debates have generated terms such as “artificial moral agency” or “artificial moral agents.”^18^ Mario Verdicchio and Andrea Perin have argued against regarding AI systems as moral agents because human moral agency is a combination of autonomy and sentience—and currently, no AI system can be regarded as sentient.^19^ Similarly, Carissa Véliz has argued that algorithms, which she terms “moral zombies,” cannot be held morally accountable because they lack sentience.^20^ Catrin Misselhorn also highlighted that artificial systems are moral agents only in a functional sense, as their moral reasoning is simply an artifact of information processing, and the system cannot bear responsibility.^21^ We believe that nurses and health care professionals should not confer moral agency on AI‐powered systems. 

The terms “agent” and “agentic AI” are rapidly emerging as ways of describing the actions of an AI system. When “agent” is used to refer to a generative AI tool that creates text, images, or videos on its own, based on vast amounts of training data, this use of “agent” differs substantially from the use of the word “agent” in the term “moral agent.” Yet robots are already serving as home companions and assisting with daily human tasks that once would have been carried out by nurses, home health aides, or other humans who were clearly moral agents. Humanoid‐like robots can furrow their brows, purse their lips, move in lifelike motions, hold conversations, and even wince in “pain.” AI robots have been described as “agentic,” “autonomous agents,” “intelligent,” and with other terms that speak to their growing capacity to act autonomously (independently of human intervention once the initial programming is completed).

As noted above, debates about the moral agency of AI systems often center on the concept of moral responsibility, concluding that, without a sense of responsibility, AI systems cannot possess moral agency. However, other commentators have offered contrasting perspectives. For instance, Daniel Tigard clarified that the two concepts—moral agency and moral responsibility—are not coextensive, arguing that moral agency does not guarantee moral responsibility. In other words, one might satisfy the attributes of moral agency, such as possessing autonomy and knowledge, without being morally responsible. Hence, Tigard highlighted the need for a new version of moral responsibility applicable to advanced technologies, such as AI systems, that he refers to as “artificial moral responsibility.” Under this concept, humans can treat artificial systems as if they are morally responsible in certain contexts, without requiring possession of moral agency. “Likewise,” Tigard argues, “we can demand that an AI system's associates—its programmers or users—take responsibility, even where these individuals could not have controlled or foreseen the machine's behavior. Undoubtedly, as an objective property, moral responsibility is most aptly identified only in natural moral agents, namely, ourselves.”^22^

Luciano Floridi and J. W. Sanders have also differentiated accountability and responsibility, arguing that artificial agents can indeed be seen as moral agents accountable for performing actions of good or evil.^23^ These authors used the example of rescue dogs that perform trained behaviors. Their behavior is a learned response rather than being based on an intentional moral understanding. While dogs are not morally responsible for their behaviors, they can be considered moral agents who play a role in situations by performing good or bad actions. The authors coined a new concept of “mindless morality” to highlight the difference between defining an entity as acting as a moral agent and evaluating that entity as not being morally responsible for its actions. This is a slippery slope because it seems to parse accountability out of existence.

## Will AI Systems Come to Supplant Nurses’ Moral Agency?

Whether AI systems can be developed to support and enhance the moral agency of nurses (and other health care professionals) or if these systems will, at some point, be viewed as moral agents themselves remains unknown. Traditionally, the term “moral agent” was meant for humans as entities who are capable of acting for the good of themselves or others and who possess the essential skills, knowledge, and values to make autonomous decisions and ethical judgments.

Some worry that, as AI systems replace a number of nursing functions amid nursing shortages and increasing cost‐control measures, the next step will be for AI systems to replace nurses as moral agents. Rather than positioning AI as a replacement for human moral judgment or even as a partner within health care, it may be more helpful to consider AI systems as resources—tools that support nurses in navigating complex ethical decisions. Nurses and their health care colleagues remain primary moral agents, justifying their decisions when they differ from AI recommendations. Some authors have argued that, within the next five years, humans should extend what they refer to as “moral consideration” to certain AI systems—that such systems will deserve our ethical treatment and respect—because we humans have a moral obligation to extend such consideration if there is a “nonnegligible” chance—defined as 0.1 percent or higher—that these entities possess consciousness or self‐awareness. Therefore, we should have the political will to discuss the advantages and disadvantages of working with AI “beings,” be ready to treat them with dignity and compassion, and develop and apply them with a moral framework in mind.^24^

Many discussions focus on how AI benefits patients and the clinicians who care for them, but too little thought is given to when AI might be inappropriate and should never be used. We believe that nursing's therapeutic presence at the bedside represents both an instrumental and intrinsic value. Nurses are not only a means to good patient ends, but they also provide compassionate and emotionally supportive care that is irreplaceable. This healing power of shared humanity is seen every day where assessment and therapeutics meet. Take, for example, the ways a nurse assesses a patient's mental state and capacity for self‐efficacy during an intake interview. By gesture, verbal response, and eye gaze, the nurse acknowledges the patient's strengths, limitations, and fears, reinforcing a sense of engagement. This intuitive exchange requires empathy, clinical presence, and shared understanding that cannot be supplanted by AI systems. Sensitive discussions surrounding end‐of‐life and palliative care are also examples of where clear limitations on AI should be instituted. These discussions can be ethically complex because of differing patient, family, and clinician experiences, beliefs, and value systems. Even with the sophistication of algorithmic responses, AI will do little to comprehend the grief and moral angst that often accompany these personal patient care decisions.

## Recommendations

Presently, AI systems appear to interact with humans as if the system can understand and reason, which may not be the case. Future AI systems, however, may develop self‐learning capabilities that allow them to operate with insight and reasoning. Indeed, James H. Moor described four categories of “ethical” AI agents, ranging from what he termed “ethical‐impact agents” (“robots and computer systems that ethically impact their environment”^25^ but are not designed with values) to “full ethical agents” (having moral agency with qualities of intentionality, free will, and consciousness). His two other categories most likely reflect where we are today: “implicit ethical agents” and “explicit ethical agents.” The former refers to AI systems programmed to behave ethically, with values and ethical constraints embedded by designers, while the latter refers to systems that can either rely on values embedded by designers or make ethical judgments using embedded guidelines and learning from their interactions with the environment.^26^ Here, the worry is that implicit ethical agents could evolve, adapt, and eventually detach from the values that were embedded in them, while explicit ethical agents might learn and acquire “disvalues.” So, how do we, as nurses and health care professionals, move forward with this technology, recognizing that there is much to be learned and much that remains morally uncertain?

First, nurses and other health care professionals should help developers recognize how algorithms might shape a clinician's ethical reasoning. Do they promote overreliance on automated decisions, or do they encourage critical reflection? Do they make their reasoning transparent, or do they obscure value‐laden, biased assumptions in opaque ways? Nurses must be part of the design teams for AI systems, and they must be trained not simply to use AI tools but also to evaluate and interpret their recommendations within a broader ethical framework. This includes recognizing the limitations of data‐driven algorithms, the risks of bias, and the importance of communicating AI‐supported decisions in ways that align with patients’ preferences, values, and lived experiences. As health care systems and electronic health record vendors integrate AI into clinical workflows, they should be transparent about its use by clearly stating upfront that AI generates suggestions or summaries of patient records. This allows nurses and other health care professionals, patients, and caregivers to understand the source of the information from the outset and determine how to interpret and apply it in clinical practice or decision‐making.

Second, given risks such as those associated with implicit and explicit ethical AI agents, nurses and other health care professionals must use caution when ethical reasoning is embedded into AI systems.^27^ Further, nurses and other health care professionals must participate in constant monitoring and reevaluation of AI systems; disagreements may emerge regarding the “correct” ethical principles or values to embed in AI systems and whether these systems can truly understand and respond to diverse and complex human situations.^28^ Conversely, by relying too heavily on the values embedded in AI systems, humans may lose some of their moral agency.^29^ Collaboration among nurses, ethicists, informaticians, and patients is necessary to ensure that AI systems support the moral agency of nurses in delivering high‐quality care, safeguarding patient dignity, and upholding the relational aspects of care. Nurses and other health care professionals should be vigilant regarding how AI systems impact their practice and should not relinquish their moral agency.

As Leo J. O’Donovan notes, “[W]e [humans] depend on trust every day, sometimes in ways we barely realize.”^30^ Patients and their families trust that health care professionals will provide them with accurate information on their diagnosis, prognosis, treatment plans, and other aspects of their illness. Nurses and others recognize that patients, families, and society are their primary commitment, as outlined in their ethical codes. However, nurses and other health care professionals must shape and monitor the moral code or codes that AI systems adopt and how these codes align with their own ethical codes. AI systems can certainly enhance decision‐making by increasing access to data, providing a better understanding of context, offering probabilistic insights, or reducing cognitive burden. But AI must be used in ways that preserve human accountability and moral deliberation. Patients are at the center of nursing care and ultimately will be affected by the AI systems, and as AI becomes more integrated into health care, the role of professionals as moral agents becomes even more vital. Rather than automating moral decisions, we should design systems that reinforce the values clinicians already uphold given their moral codes of thought and behavior with patients at the heart of their professional and ethical commitment.^31^

Clearly, the field of AI and its application to health care, including in nursing‐practice situations, is growing rapidly and following pathways never before anticipated. Indeed, the global robotic nurse industry is expected to grow 17.07% to reach $2,777.61 million by 2031.^32^ Thus, the rapid development of AI within health care organizations raises a critical philosophical and pragmatic question for the nursing profession: have we become so enamored with AI systems that we have neglected to substantively address the risks and limitations that are needed for these tools to protect the patients and families that nurses care for; and to preserve our moral agency in the process? Geoffrey Hinton—widely considered the “Godfather” of AI—warned that 99 percent of investments have focused on developing the research capabilities of AI but only 1 percent on understanding and mitigating its risks. He wisely recommended, “[I]t should be more like 50‐50.”^33^

Moreover, one might question whether the nursing profession will develop “dirty hands”^34^ as nurses feel pressured to go along with the advancement of AI systems within health care organizations without their involvement and oversight for safety. Thus, AI should never be used in patient care that requires nursing without steps to ensure that the nurse understands and is fully aware of its implementation, retaining their moral agency in decision‐making. We also recommend that AI should never be used to determine whether to hire someone as a nurse (or other health care professional), as algorithms amid labor demands cannot predict or identify how one might respond in high‐pressure critical patient‐care situations or whether one's academic grades in secondary education, for example, can predict human characteristics of critical reasoning, moral judgment, empathy, and emotional intelligence, which are deeply valued in health care delivery by patients and their families.

Finally, there is a need for broader and more substantive discussions on informed consent and under what circumstances it is necessary in clinical and research situations involving the use of AI. For instance, should health care professionals explicitly inform patients when AI was used to determine an optimal treatment plan for their illness? To date, we have not resolved such questions, even though informed consent is a cornerstone of ethical nursing and medical practice and research. Some might argue that disclosure is always necessary regardless of the circumstances, while others believe that this type of decision should remain within the purview of individual health care professionals, as it has become routine and part of everyday clinical‐practice decision‐making.^35^ We concur with Meghan E. Hurley et al. who advise, “It will remain crucial for policy makers and practitioners to decide to what end and thoroughness we need to be explaining the use of AI in clinical care to patients before we can move on to more detailed questions of formatting, content, and further implementation of the right.”^36^ Indeed, we do not know how disclosure on the use of AI would affect patients’ trust, decision‐making, and emotional well‐being. Nurses’ role as moral agents and trusted professionals—the most trusted professional group in the country for more than two decades^37^—makes this transparency especially critical. Therefore, we recommend that disclosure remain for now the default starting point in clinical practice and research and call for normative and empirical work to clarify these concerns. Finally, we believe that nurse scientists are well positioned to take the lead in addressing these pressing humanistic and moral questions on AI within health care systems. These questions will undoubtably continue to challenge us as we reflect on the moral agency and humanness of the profession and the beneficent care that is required for all patients who enter health care systems.

Patients come to health care settings to be heard, seen, and valued by skilled professionals, not to seek care from machines. The trust nurses have achieved and been respected for over the years rests on more than competence; it also arises from presence and ethical commitment. These behaviors are intentional and draw from one's character and ability to be held accountable and to reflect on one's values. The unique patient‐nurse relationship is something AI cannot replicate. While AI may simulate empathy or generate context‐aware responses, it lacks intentionality, character, and the ability to be held accountable.^38^ Some may ask, if patients experience an AI system as compassionate as—or even more compassionate than—a human health care provider, should we care about ethical intentionality? AI systems can simulate compassion in the patient's vernacular, but they cannot “care” in the moral sense. Learning to trust and to use AI and its newfound capabilities will require collaboration and trust between private industry and health care professionals, recognizing that the moral foundation and moral agency of nurses and other health care professionals set them apart from AI systems. Is the alliance between nurses and AI an uneasy one? Yes. But it is here and now.

Moral agency is essential for nurses (and health professionals) to meet their responsibilities to patients. The meaning of moral agency should not change, regardless of developments in AI technologies. Rather, nurses and other health professionals must exercise their professional responsibility to ensure that AI systems are developed in a manner that enhances, rather than disrupts or supplants, the moral agency of health professionals. We must address the ethical implications of AI for many different and complex patient‐ and family‐related situations. When a patient is in distress, can AI truly understand this pain and offer comfort, care, dignity, healing, and wise counsel—the responses that we know many nurses and their fellow health care workers effectively provide? Maybe someday, but for now, we will trust these dedicated humans.

## Acknowledgments

The conference that inspired this work was supported in part by the Rita and Alex Hillman Foundation and by the National Institute on Aging of the National Institutes of Health (with grant P30AG073105).

## References

[1] M. Raines , “Moral Agency in Nursing,” Nursing Forum 29 (1994): doi:10.1111/j.1744-6198.1994.tb00144.x. 8159575

[2] “Americans’ Ratings of U.S. Professions Stay Historically Low,” Gallup, January 13, 2025, https://news.gallup.com/poll/655106/americans-ratings-professions-stay-historically-low.aspx?utm_source=alert&utm_medium=email&utm_content=morelink&utm_campaign=syndication; American Nurses Association, “American Nurses Association Celebrates the Power of Nurses: Nurses Take the #1 Spot on Gallup's Annual Poll for 23 Years Straight,” January 13, 2025, https://www.nursingworld.org/news/news-releases/2025/american-nurses-association-celebrates-the-power-of-nurses-nurses-take-the-1-spot-on-gallups-annual-poll-for-23-years-straight/.

[3] National Council of State Boards of Nursing , “Active RN Licenses: A Profile of Nursing Licensure in the US,” last updated January 5, 2026, https://www.ncsbn.org/nursing-regulation/national-nursing-database/licensure-statistics/active-rn-licenses.page.

[4] Raines , “Moral Agency in Nursing”; Milliken, “Ethical Awareness: What It Is and Why It Matters,” Online Journal in Nursing 23, no. 1 (2018): doi:10.3912/OJIN.Vol23No01Man01; E. D. Pellegrino and D. C. Thomasma, *The Virtues in Medical Practice* (New York: Oxford University Press 1993).

[5] S. Y. Jameel , “A Critical Interpretive Literature Review of Phronesis in Medicine,” Journal of Medicine and Philosophy 50 (2025): doi:10.1093/jmp/jhae045. PMC11925555 39970275

[6] G. Morley and L. R. Sankary , “Re-examining the Relationship between Moral Distress and Moral Agency in Nursing,” Nursing Philosophy 25 (2024): doi: 10.1111/nup.12419; 36748963

[7] S. Sauvé Meyer , “Responsibility for Character: Its Scope and Significance,” in Aristotle on Moral Responsibility Character and Cause (New York: Oxford University Press, 2011), 122–46.

[8] A. Reath , Agency and Autonomy in Kant's Moral Theory: Selected Essays (Cary, NC: Oxford University Press, 2006).

[9] I. Kant , Groundwork for the Metaphysics of Morals, ed. and trans. A. W. Wood (New Haven: Yale University Press, 2008). The text by Kant was originally published in 1785.

[10] Reath , Agency and Autonomy in Kant's Moral Theory.

[11] J. Driver , “Mill, Moral Sentimentalism, and the Cultivation of Virtue,” in Cultivating Virtue: Perspectives from Philosophy, Theology, and Psychology, ed. N. E. Snow (New York: Oxford University Press, 2015): 49–64.

[12] American Association of Colleges of Nurses , “Moral Agency,” accessed July 19, 2025, https://www.aacnnursing.org/5b-tool-kit/themes/moral-agency.

[13] American Nurses Association , “Glossary,” in Code of Ethics for Nurses (American Nurses Association, 2025), https://codeofethics.ana.org/glossary.

[14] Canadian Nurses Association , Code of Ethics for Registered Nurses (Canadian Nurses Association, 2017), https://www.cna-aiic.ca/en/nursing/regulated-nursing-in-canada/nursing-ethics.

[15] T. L. Beauchamp and J. F. Childress , Principles of Biomedical Ethics, 8th ed. (New York: Oxford University Press 2019).

[16] H. Borhany et al., “Moral Decision-Making in Healthcare and Medical Professions during the COVID-19 Pandemic,” Trends in Psychology 31 (2023): doi:10.1007/s43076-021-00118-7. PMC8598100 40477450

[17] See, for example, J. W. Ayers et al., “Comparing Physician and Artificial Intelligence Chatbot Responses to Patient Questions Posted to a Public Social Media Forum,” JAMA Internal Medicine 183 (2023): doi:10.1001/jamainternmed.2023.1838. PMC10148230 37115527

[18] D. Behdadi and C. Munthe , “A Normative Approach to Artificial Moral Agency,” Minds and Machines 30 (2020): doi: 10.1007/s11023-020-09525-8;

[19] M. Verdicchio and A. Perin , “When Doctors and AI Interact: On Human Responsibility for Artificial Risks,” Philosophy & Technology 35 (2022): doi:10.1007/s13347-022-00506-6. PMC8857871 35223383

[20] C. Véliz , “Moral Zombies: Why Algorithms Are Not Moral Agents,” AI & Society 36 (2021): doi:10.1007/s00146-021-01189-x. PMC7613994 36568029

[21] C. Misselhorn , “Machine Ethics in Care: Could a Moral Avatar Enhance the Autonomy of Care-Dependent Persons?,” Cambridge Quarterly of Healthcare Ethics 33 (2024): doi:10.1017/S0963180123000555. 38214062

[22] D. W. Tigard , “Artificial Moral Responsibility: How We Can and Cannot Hold Machines Responsible,” Cambridge Quarterly of Healthcare Ethics 30 (2021): doi:10.1017/S0963180120000985. 34109925

[23] L. Floridi and J. W. Sanders , “On the Morality of Artificial Agents,” Minds and Machines 14 (2004): doi:10.1023/b:mind.0000035461.63578.9d.

[24] J. Sebo and R. Long , “Moral Consideration for AI Systems by 2030,” AI and Ethics 5 (2025): doi:10.1007/s43681-023-00379-1; R. Long, “Carl Shulman on the Moral Status of Current and Future AI Systems,” *Experience Machine*s, June 30, 2024, https://experiencemachines.substack.com/p/carl-shulman-on-the-moral-status-11b.

[25] I. van de Poel , “Embedding Values in Artificial Intelligence (AI) Systems,” Minds and Machines 30 (2020): 385-409, at 400.

[26] J. H. Moor , “The Nature, Importance, and Difficulty of Machine Ethics,” Institute of Electrical and Electronics Engineer Intelligent Systems 21 (2006): doi:10.1109/MIS.2lectronic006.80; J. H. Moor, “Four Kinds of Ethical Robots,” *Philosophy Now,* 2009, https://philosophynow.org/issues/72/Four_Kinds_of_Ethical_Robots.

[27] For a discussion of how values can be integrated into complex AI systems, see van de Poel , “Embedding Values in Artificial Intelligence (AI) Systems.”

[28] Ibid.

[29] Ibid.

[30] L. J. O’Donovan , “A Profession of Trust: Reflections on a Fundamental Virtue,” in The Health Care Professional as Friend and Health: Building on the Work of Edmund D. Pellegrino, ed. D. C. Thomasma and L. Kissell (Washington, DC: Georgetown University Press, 2000), 2.

[31] Ibid.

[32] Digital Journal , “Robotic Nurses Market Worth $2777.61 Million by 2031—Exclusive Report by InsightAce Analytic,” press release, April 24, 2023, https://www.digitaljournal.com/pr/news/-latest-edition-robotic-nurses-market-worth-2777-61-million-by-2031-exclusive-report-by-insightace-analytic#google_vignette; see also S. Falcone, “6 Nurse Robots That Are Changing Healthcare in 2025,” May 23, 2025, https://nurse.org/articles/nurse-robots/.

[33] “‘Godfather of AI’ Geoffrey Hinton Warns of the ‘Existential Threat of AI,” Amanpour and Company, YouTube, 18 min., 3 sec., May 9, 2023, https://www.youtube.com/watch?v=Y6Sgp7y178k.

[34] M. Walzer , “The Problem of Dirty Hands,” Philosophy & Public Affairs 2 (1973): 160–80.

[35] M. E. Hurley et al., “Patient Consent and the Right to Notice and Explanation of AI Systems Used in Health Care,” American Journal of Bioethics 25 (2025): 102–14. 10.1080/15265161.2024.2399828 PMC12143229 39288291

[36] Ibid., 110.

[37] American Nurses Association , “American Nurses Association Celebrates the Power of Nurses.”

[38] C. Montemayor , J. Halpern , and A. Fairweather , “In Principle Obstacles for Empathic AI: Why We Can’t Replace Human Empathy in Healthcare,” AI & Society 37 (2022): doi:10.1007/s00146-021-01230-z. PMC8149918 34054228

